# Activation of GPR40 attenuates neuroinflammation and improves neurological function via PAK4/CREB/KDM6B pathway in an experimental GMH rat model

**DOI:** 10.1186/s12974-021-02209-9

**Published:** 2021-07-18

**Authors:** Jie Xiao, Tao Cai, Yuanjian Fang, Rui Liu, Jerry J. Flores, Wenna Wang, Ling Gao, Yu Liu, Qin Lu, Lihui Tang, John H. Zhang, Hongwei Lu, Jiping Tang

**Affiliations:** 1 Department of Emergency, The Third Xiangya Hospital of Central South University, 138 Tongzipo Road, Changsha, Hunan 410013 People’s Republic of China; 2 Department of Physiology and Pharmacology, Loma Linda University School of Medicine, Loma Linda, California, 92354 USA; 3 Department of Neurosurgery, The Third Xiangya Hospital of Central South University, 138 Tongzipo Road, Changsha, Hunan 410013 People’s Republic of China; 4 Departments of Anesthesiology, Neurosurgery and Neurology, Loma Linda University School of Medicine, Loma Linda, California, 92354 USA; 5 Center for Experimental Medicine, The Third Xiangya Hospital of Central South University, 138 Tongzipo Road, Changsha, Hunan 410013 People’s Republic of China

**Keywords:** GPR40, Germinal matrix hemorrhage, Microglia, Neuroinflammation

## Abstract

**Background:**

Germinal matrix hemorrhage (GMH) is defined by the rupture of immature blood vessels in the germinal matrix, where subsequent hemorrhage enters the subependymal zone and the cerebral lateral ventricles. The consequent blood clot has been identified as the causative factor of secondary brain injury, which triggers a series of complex parallel and sequential harmful mechanisms, including neuroinflammation. The orphan G-protein-coupled receptor 40 (GPR40), a free fatty acid (FFA) receptor 1, has been shown to exert anti-inflammatory effects when activated and improved outcomes in animal models of stroke. We aimed to investigate the anti-inflammatory effects of GPR40 and its underlying mechanisms after GMH.

**Methods:**

GMH model was induced in 7-day-old rat pups by an intraparenchymal injection of bacterial collagenase. GPR40 agonist, GW9508, was administered intranasally 1 h, 25 h, and 49 h after GMH induction. CRISPR targeting GPR40, PAK4, and KDM6B were administered through intracerebroventricular injection 48 h before GMH induction. Neurologic scores, microglia polarization, and brain morphology were evaluated by negative geotaxis, right reflex, rotarod test, foot fault test, Morris water maze, immunofluorescence staining, Western blots, and nissl staining respectfully.

**Results:**

The results demonstrated that GW9508 improved neurological and morphological outcomes after GMH in the short (24 h, 48 h, 72h) and long-term (days 21–27). However, the neuroprotective effects of treatment were abolished by GW1100, a selective GPR40 antagonist. GW9508 treatment increased populations of M2 microglia and decreased M1 microglia in periventricular areas 24 h after GMH induction. GW9508 upregulated the phosphorylation of PAK4, CREB, and protein level of KDM6B, CD206, IL-10, which was also met with the downregulation of inflammatory markers IL-1β and TNF-α. The mechanism study demonstrated that the knockdown of GPR40, PAK4, and KDM6B reversed the neuroprotective effects brought on by GW9508. This evidence suggests that GPR40/PAK4/CREB/KDM6B signaling pathway in microglia plays a role in the attenuation of neuroinflammation after GMH.

**Conclusions:**

In conclusion, the present study demonstrates that the activation of GPR40 attenuated GMH-induced neuroinflammation through the activation of the PAK4/CREB/KDM6B signaling pathway, and M2 microglia may be a major mediator of this effect. Thus, GPR40 may serve as a potential target in the reduction of the inflammatory response following GMH, thereby improving neurological outcomes in the short- and long-term.

## Background

Germinal matrix hemorrhage (GMH) is the most common neurological disorder of preterm infants, which is associated with a high rate of disability and mortality [1]. GMH results from the rupture of immature blood vessels in the subependymal tissue that leads to primary and secondary brain injury, which results in hydrocephalus, epilepsy, and developmental delay [2]. Microglia have been attributed to play a primary role in the mediation of neuroinflammation, which has been shown to contribute to post-hemorrhagic tissue destruction causing morphological and functional impairments [3]. Therefore, the inhibition of the neuroinflammation could be a potential therapeutic strategy for patients with GMH.

Microglia are resident macrophages of the central nervous system, which have been shown to contribute to the acute inflammatory response after GMH and other hemorrhagic brain diseases [4, 5]. After brain hemorrhage, microglia cells are activated and polarized into two distinct phenotypes which are defined as the classically activated (M1) phenotype and the alternatively activated microglia (M2) phenotype [6]. M1 phenotypic microglia have been shown to be pro-inflammatory, where the production of inflammatory cytokines (e.g., IL-1β, IL-12, tumor necrosis factor-α (TNF-α)) have shown to cause brain injury [7]. By contrast, M2 phenotypic microglia contribute to CNS repair, as various studies have demonstrated that M2 microglia play a vital role in the clearance of cell debris, resolution inflammation, the phagocytosis of RBC, and releases a plethora of trophic factors to promote brain recovery (e.g., IL-10, Arginase 1, CD206, Ym1, Fizz1) [8–13]. Therefore, the polarization of microglia from M1 to M2 state may provide a potential therapy for GMH.

G-protein-coupled receptor 40 (GPR40), also known as free fatty acid (FFA) receptor 1, belongs to class A G-protein-coupled receptors (GPCRs) and has a high affinity for medium- and long-chain saturated or unsaturated free fatty acid. GPR40 is located in various diverse organs such as the brain, pancreas, spinal cord, and heart [14–18]. Several studies have shown that the activation of GPR40 has had beneficial anti-inflammatory effects and provided neuro-protection after CNS injury [19–21]. Recent studies have shown that Eicosapentaenoic acid (EPA) elicits anti-inflammatory actions through the upregulation of GPR40 after acute cerebral infarction [22]. The activation of GPR40 potentiated the phosphorylation of p21-activated kinases4 (PAK4) in mouse islet cells [23]. Moreover, phosphorylated PAK4 has been shown to mediate the phosphorylation of CAMP-response element-binding (CREB), which promoted the protection of dopaminergic neurons in a rat model of PD and preserved motor function [24]. Recent studies also revealed that CREB induces the gene expression of histone H3 lysine 27 (H3K27) demethylase (KDM6B), which plays a role in the regulation of inflammatory gene expression and regulates phenotype switching of microglia from the M1 to M2 state [25–27]. Additionally, inhibition of KDM6B resulted in the attenuation of M2 microglia population, exacerbated the inflammatory response, and promoted M1 microglia [28, 29]. However, GPR40’s role and its mechanism of action after GMH remain to be elucidated.

In this study, we demonstrated that GPR40 agonism attenuated pro-inflammatory cytokines, through the promotion M2-like microglia which improved short- and long-term neurological deficits in neonatal rat models of GMH through the PAK4/CREB/KDM6B signaling pathway.

## Materials and methods

### Animals

All experiments were approved by the Institutional Animal Care and Use Committee at Loma Linda University and in compliance with the NIH Guidelines for the use of animals in neuroscience research. Two hundred and thirty-five P7 Sprague-Dawley neonatal pups (weight = 12–14 g, Harlan, Livermore, CA) were used.

### Germinal matrix hemorrhage (GMH) model

The GMH model was induced by bacterial collagenase fusion as previously described in (Zhang et al., 2018) [30]. P7 neonatal pups were anesthetized using isoflurane (4% induction, 2% maintenance) and secured onto a neonatal stereotaxic frame. The skin along the longitudinal plane was incised exposing the bregma, which was followed by the drilling of a 1-mm burr hole into the skull at the following coordinates relative to bregma: 1.6 mm anterior and 1.5 mm right lateral. A 27-gauge needle with 0.3 U clostridial collagenase (Sigma-Aldrich, MO) was inserted at a depth of 2.7 mm from the dura through the burr hole and infused (1 μl/min) using a 10 μl Hamilton syringe (Hamilton Co., Reno, NV, USA) which was guided by a micro-infusion pump (Harvard Apparatus, Holliston, MA). The syringe was kept in place for an additional 5 min to avoid backflow. Lastly, the syringe was withdrawn at a speed of 0.5 mm/min, bone wax was used to close the burr hole, and the incision line was sutured. The animals were then placed onto a 37°C heated blanket for recovery and then reunited with the dam. Sham animals were operated on with needle insertion without collagenase infusion.

### Experimental design

The current study was performed in four separate experiments.

#### Experiment 1: Time course of GPR40, p-PAK4, PAK4, KDM6B, CD16, CD206, and cellular localization of GPR40, KDM6B in P7 rat pups

Forty-two P7 rat pups were randomly divided into seven groups (n = 6 per group): Sham and GMH (3 h, 6 h, 12 h, 24 h, 3 days, 7 days). The whole brains of six rats from each group were collected for western blotting to determine the protein levels of GPR40, p-PAK4, PAK4, KDM6B, CD16, and CD206. The double immunofluorescence staining of GPR40 or KDM6B were used in the sham group and GMH (24 h) group (n = 3 per group) to evaluate the cellular localization of GPR40 or KDM6B in microglia, neurons, and astrocytes.

#### Experiment 2: The effects of GPR40 activation on short-term outcomes after GMH

Thirty P7 neonatal rat pups were randomly assigned to five groups (n = 6 per group): Sham; GMH + vehicle (10% dimethyl sulfide), GMH + GW9508 (0.84 mg/kg), GMH+ GW9508 (2.5 mg/kg), and GMH+ GW9508 (7.5 mg/kg). GW9508 was administrated intranasally (i.n.) 1 h, 25 h and 49 h after GMH. Neurological behavior tests were measured at 24 h, 48 h, and 72 h after GMH. Randomly, fifteen more rat pups were divided into three experimental groups with n = 5 for each group: Sham, GMH + vehicle (10% dimethyl sulfide), and GMH + GW9508 (best dose). These three groups were evaluated for microglia polarization by double immunofluorescence staining. The best dose of GW9508 was chosen based on the short-term neurological outcomes for the long-term outcome and mechanism experiments.

#### Experiment 3: The effect of GPR40 on long-term outcomes after GMH.

Sixty animals were randomly subjected to four groups (n = 15 per group): Sham, GMH + vehicle (10% dimethyl sulfide), GMH + GW9508, and GMH + GW9508 + GW1100 for evaluation of long-term neurological scores and histological result. GW9508 was administrated intranasally (i.n.) 1 h, 25 h, and 49 h after GMH. GW1100 was administrated intranasally (i.n.) 1 h before, and 23 h, 47 h after GMH. The rotarod and foot fault tests were performed on day 21 post-GMH. Morris water maze test was carried out on days 22–27 following GMH. Brain tissues were then obtained for Nissl staining to evaluate morphological changes.

#### Experiment 4: Mechanism study (GPR40/PAK4/CREB/KDM6B signaling pathway)

To investigate the potential molecular mechanism of GPR40 activation, 42 rats were randomly divided to seven groups (n = 6 per group): Sham, GMH + vehicle (10% dimethyl sulfide, i.n.), GMH + GW9508, GMH + GW9508 + scrambled CRISPR, GMH + GW9508 + GPR40 K.O. CRISPR, GMH + GW9508 + PAK4 K.O. CRISPR, GMH + GW9508 + KDM6B K.O. CRISPR. Furthermore, to determine the CRISPR/Cas9 editing efficiency, another 40 rats (n = 5 for each group) were randomly allocated into eight groups: naive + Scrambled CRISPR, naive + GPR40 K.O. CRISPR, naive + PAK4 K.O. CRISPR, naive + KDM6B K.O. CRISPR, GMH + scrambled CRISPR, GMH + GPR40 K.O. CRISPR, GMH + PAK4 K.O. CRISPR, GMH + KDM6B K.O. CRISPR. The whole brain was used for WB detection after neurological test.

### Drug administration

#### Intranasal drug administration

Intranasal drug administration was conducted at 1 h, 25 h, and 49 h after GMH induction as described in (Zhang et al., 2017) [31]. A volume of 10 μl was administered of either 10% dimethyl sulfide (vehicle) or GW9508 (G9797, Sigma Aldrich, MO) at three different dosages (0.84 mg/kg, 2.5 mg/kg, 7.5 mg/kg). The dosage of GW9508 was adapted from previous literature [32].

#### Intracerebroventricular drug administration

Intracerebroventricular (ICV) drug administration was delivered as previously described in (Flores et al., 2016) [3]. Briefly, the site of i.c.v injection was at the following coordinates relative to bregma: 1.0 mm posterior and 1.0 mm lateral. A 10 μL Hamilton syringe (Microliter 701, Hamilton Company, Reno, NV, USA) was inserted through the burr hole into the left lateral ventricle at a depth of 1.8 mm. GPR40 CRISPR (Santa Cruz Biotechnology, Dallas, TX, USA), PAK4 CRISPR (Santa Cruz Biotechnology, Dallas, TX, USA), KDM6B CRISPR (Santa Cruz Biotechnology, Dallas, TX, USA), or Scramble CRISPR (Santa Cruz Biotechnology, Dallas, TX, USA) was infused into the ipsilateral ventricle 48 h prior to GMH induction. The needle was kept in place for 5 min and was slowly withdrawn over 3 min to prevent backflow.

### Neurological performance

#### Short-term neurobehavioral examination

Negative geotaxis and right reflex were performed at 24 h, 48 h, and 72 h post-GMH as previously reported in (Feng et al., 2019) [33]. Negative geotaxis was conducted 3 times per pup, and the average time was obtained. Righting reflex was performed 3 times per rat pup, and the tests’ average time was obtained. These two examinations were employed to evaluate the neurological function in neonate rat pups.

#### Long-term neurobehavioral examination

1. Rotarod test

Twenty-eight days post-ictus, rotarod test was conducted to assess the motor impairment as previously described in (Keleb et al., 2017) [34]. The duration of animals on the accelerating rotarod was recorded.

2. Foot fault test

Foot fault test was employed to assess changes in locomotor function [35]. The numbers of foot faults were recorded for 60s for each rat using a video recording device.

3. Morris water maze

The Morris water maze was used to evaluate spatial learning and memory at days 23–27 after GMH [36]. The water maze is made up of a plastic tub with an adjustable stand used as an escape for rats. Briefly, the rats were first trained to find the platform (cued test), which was then followed by the submerging of the platform (special test) where the rats would search for the platform in a duration of 60 s. On the last day, the platform was removed and the rats were tested for memory reference (probe trial) for a duration of 60 s.

### Immunofluorescence

Immunofluorescence was conducted as previously reported in (Pan et al., 2014, Pan et al., 2015) [37, 38]. Animals were anesthetized until unresponsive to stimuli, followed by trans-cardiac perfusion with ice-cold PBS. After the brains were removed, they were fixed in 10% formalin overnight at 4°C, then transferred to 30% sucrose until they sunk to the bottom. The brains were embedded and frozen at −80°C. Samples were segmented into 10μm coronal slices. Next, the brain slices were incubated at 4°C overnight with primary antibodies: rabbit anti-GPR40 (1:50, PA5-75351, Thermo Fisher Scientific, MA, USA), rabbit anti-KDM6B (1:50, PA5-72751, Thermo Fisher Scientific, MA, USA), rabbit anti-CD11b (1:50, ab133357, Abcam, MA, USA), rabbit anti-CD206 (1:50, sc-58986, Santa Cruz Biotechnology, CA, USA), rabbit anti-CD16 (1:50, sc-58962, Santa Cruz Biotechnology, CA, USA), anti-NeuN (1:100, ab177487, Abcam, MA, USA), anti-Iba1 (1:100, ab15690, Abcam, MA, USA), and anti-GFAP (1:100, ab7260, Abcam, MA, USA). The brain slides were incubated with their respective secondary antibodies for 2 h at room temperature in a low-lit room. The cellular colocalization of GPR40 or KDM6B with NeuN, GFAP, and Iba1 was observed with the use of a fluorescence microscope (Leica DMi8, Buffalo Grove, IL). To observe microglia polarized populations, double immunofluorescence of CD16 or CD206 with CD11b was observed using the same fluorescence microscope.

### Western blot

Western blot was conducted as previously reported [39–43]. The animal’s whole brain tissue was obtained and extracted into RIPA lysis buffer (sc-24948, Santa Cruz Biotechnology, CA, USA), homogenized, and then centrifuged at 14,000 rpm at for 30 min. The equal amount of protein samples were loaded into 10% SDS-PAGE gel, then transferred to 0.22 μm nitrocellulose membranes, which were then incubated with the following primary antibodies: anti-GPR40 (1:1000, PA5-75351, Thermo Fisher Scientific, MA, USA), anti-PAK4 (1:500, sc-393367, Santa Cruz Biotechnology, CA, USA), anti-p-PAK4 (1:1000, PA5-64495, Thermo Fisher Scientific, MA, USA), anti-KDM6B (1:1000, PA5-72751, Thermo Fisher Scientific, MA, USA), anti-CREB (1:1000, 9197S Cell Signaling Technology Inc., MA, USA), anti-p-CREB (1:1000, 9198S Cell Signaling Technology Inc., MA, USA), anti-IL-1β (1:1000, 12424S Cell Signaling Technology Inc., MA, USA), anti-TNF-α (1:1000, ab6671, Abcam, MA, USA), anti-CD206 (1:1000, sc-58986, Santa Cruz Biotechnology, CA, USA), anti-IL-10 (1:1000, sc-365858, Santa Cruz Biotechnology, CA, USA), and anti-β-actin (1:3000, sc-47778, Santa Cruz Biotechnology, TX, USA) at 4 °C overnight. The membranes were then incubated with their respective secondary antibody (1:3000, Santa Cruz Biotechnology, Dallas, TX, USA and 1:5000, Millipore Sigma, Temecula, CA, USA) at room temperature for 1 h. For the quantification of western blot results, proteins and β-actin from the same sample and the same membrane corresponding to the proteins were detected. The results were normalized to the corresponding β-actin control.

### Histological volumetric study

At 28 days post-ictus, the rats were euthanized and whole brains were extracted. Nissl staining was conducted and analyzed as previously reported in [44]. Sixten-*micromet*e*r*-thick brain sections were stained with 0.5% cresyl violet (Sigma-Aldrich), and the volume of ventricular and cortical thickness was calculated using ImageJ (ImageJ 1.52, NIH, USA). The volume was calculated with the following formula: average (area of coronal section) × section interval × number of sections as described in (Flores et al., 2016) [3].

### Statistical analysis

All data are shown as the mean ± SD. GraphPad Prism (Graph Pad Software, San Diego, CA, USA) was applied to analyze the data as previously described in (Pan et al., 2020) [45]. One-way analysis of variance ANOVA with Tukey’s post hoc test was performed for comparison between multiple groups. Two-way analysis of variance ANOVA was performed to analyze the long-term neurobehavioral examinations. *p* < 0.05 was considered to be statistically significant.

## Results

### Endogenous GPR40, p-PAK4, and KDM6B were downregulated after GMH: GMH promptly increased CD16 expression, while endogenous CD206 expression was delayed until after 24 h

**Fig. 1:**
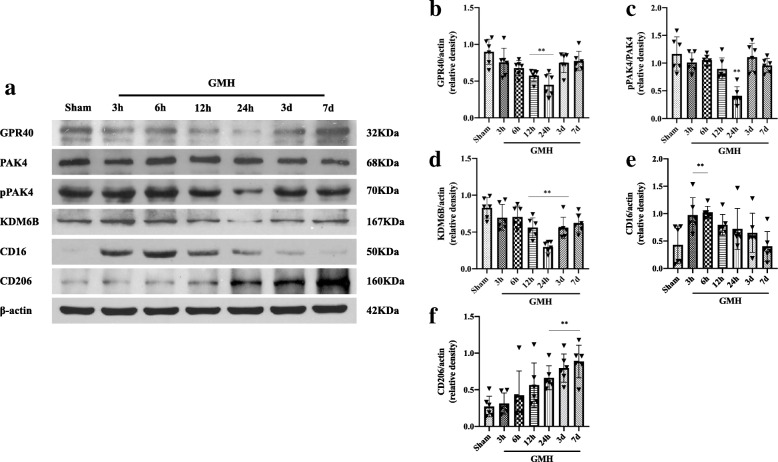
Temporal expression of GPR40, p-PAK4, PAK4, KDM6B, CD16, and CD206 in the brain after GMH. **a** The representative bands. **b**–**d** GPR40, p-PAK4, and KDM6B expression levels decreased significantly from 12 h to 24 h, reaching the lowest point at 24 h after GMH. **e** CD16 was increased and peaked rapidly at 3 h after GMH, but gradually tended to decrease afterwards. **f** The expressions of CD206 were upregulated as early as 3 h and gradually increased till 7 days after GMH. **p < 0.01 vs sham. Data are represented as mean ± SD, n = 6 for each group

A time-course study was conducted to measure the endogenous expression levels of GPR40, p-PAK4, PAK4, and KDM6B after GMH via western blot. The expression of endogenous GPR40 decreased at 3 h and reached its lowest level at 24 h after GMH (Fig. 1a, b). However, the protein expression gradually increased at 3 and 7days after GMH. Furthermore, the expressions of p-PAK4 and KDM6B demonstrated a similar downward trend in expression as GPR40 (Fig. 1a, c, and d).

**Fig. 2:**
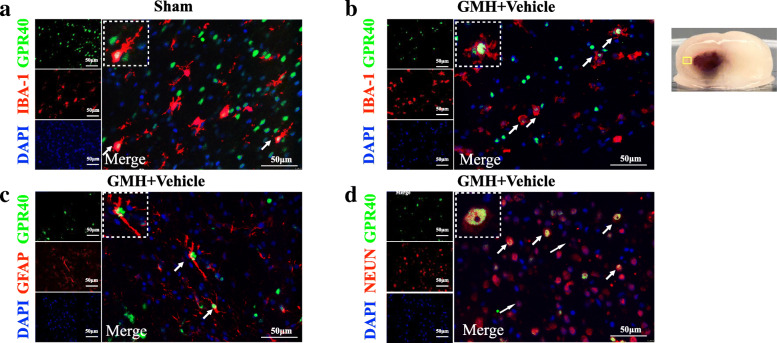
Double immunofluorescence staining of GPR40 with calcium-binding adaptor molecule 1 (Iba-1), glial fibrillary acidic protein (GFAP) and neuronal nuclei (NeuN) at 24 h after GMH. Top panel indicates the location of staining (small black box). Scale bar = 50 μm. n = 2 for each group

**Fig. 3:**
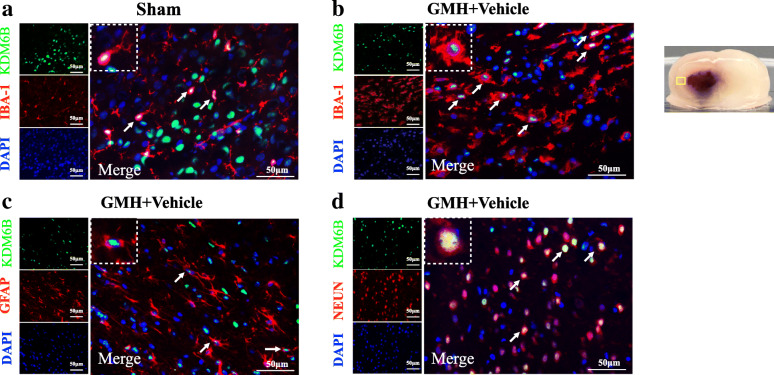
Double immunofluorescence staining of KDM6B with calcium-binding adaptor molecule 1 (Iba-1), glial fibrillary acidic protein (GFAP), and neuronal nuclei (NeuN) at 24 h after GMH. Top panel indicates the location of staining (small black box). Scale bar = 50 μm. n = 2 for each group

Furthermore, double immunofluorescence staining of GPR40 or KDM6B with Iba-1 (microglia marker), GFAP (astrocytes), or NeuN (neurons) indicated that GPR40 and KDM6B were expressed in microglia (Figs. 2a, b and 3a, b), neurons (Figs. 2c and 3c), and astrocytes (Figs. 2d and 3d) in the neonatal rat CNS. The expression of GPR40-positive microglia and KDM6B-positive microglia were greater at 24 h after GMH when compared to the Sham group.

Endogenous CD16 expression initially peaked at 3 h after GMH (Fig. 1a, e). However, the protein expression gradually decreased at 12 h after GMH induction. Furthermore, the expressions of CD206 gradually increased for 7 consecutive days after GMH (Fig. 1a, f). M1 marker CD16 and M2 marker CD206 show inverse trends of expression.

### Intranasal administration of GW9508 improved short-term neurobehavioral outcomes at 3 days after GMH

**Fig. 4:**
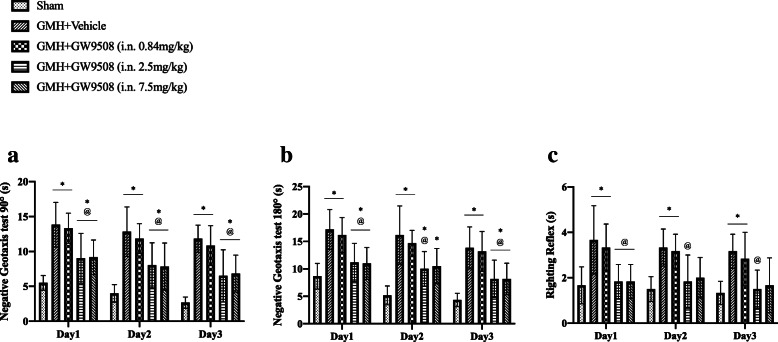
Intranasal administration of GW9508 improved short-term neurological outcomes 3 days after GMH. GMH caused neurological deficits were evaluated by negative geotaxis tests (**a**, **b**) and righting reflex (**c**), compared with the sham group. Medium dose group of GW9508 significantly improved neurological function in the first 3 days after GMH. *p < 0.05 vs sham, ^@^*p* < 0.05 vs vehicle, one-way ANOVA, Tukey test, n = 6. Mean ± SD

Both short-term behavior test (negative geotaxis and right reflex test) demonstrated significant neurobehavioral deficits in the GMH + vehicle group in the first 3 days after GMH induction when compared to the Sham group (Fig. 4a–c). GW9508 treatment at a dosage of 2.5 mg/kg and 7.5 mg/kg demonstrated significant improvements in outcomes in the negative geotaxis 90-degree test when compared with the GMH + vehicle group in the first 3 days post-GMH (Fig. 4a). Additionally, GW9508 treatment at a dosage of 2.5 mg/kg demonstrated significant improvements on the negative geotaxis 180-degree test and the right reflex test compared with the GMH + vehicle group in the first 3 days post-GMH (Fig. 4b, c). In accordance with our results, the dosage of 2.5 mg/kg was chosen as the best dosage of GW9508 and will be used to investigate the effects of GPR40 activation in the long-term and signaling pathway.

### GW9508 administration promoted proliferation of M2 microglia and inhibited M1 microglia in the periventricular area after GMH

**Fig. 5:**
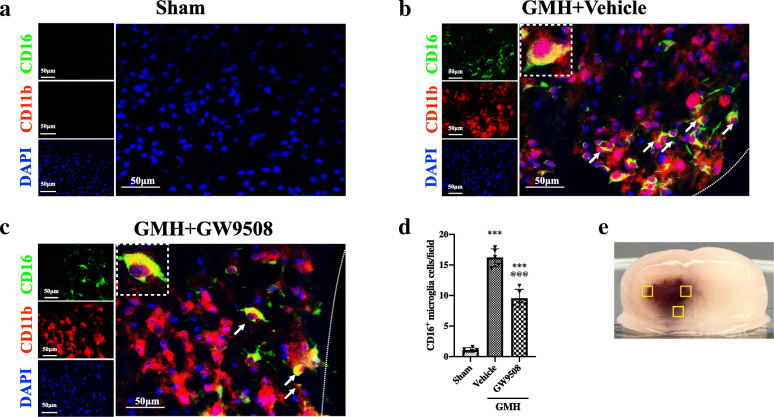
GW9508 inhibited the activation of M1 microglia surrounding the hematoma at 24 h after GMH. Double immunofluorescence staining to detect the number of M1 microglia (CD16/CD11b) in sham (**a**), GMH (**b**), and GMH + GW9508 treatment groups (**c**). **d** The number of CD16- and CD11b-positive cells per field. The error bars represent the mean ± SD. n = 5 per group, three regions surrounding the hematoma (**e**) were analyzed in each rat. ***p < 0.001 vs sham, ^@@@^*p* < 0.001 vs. GMH + vehicle. Note: peri-hematoma region is below the white broken line

**Fig. 6:**
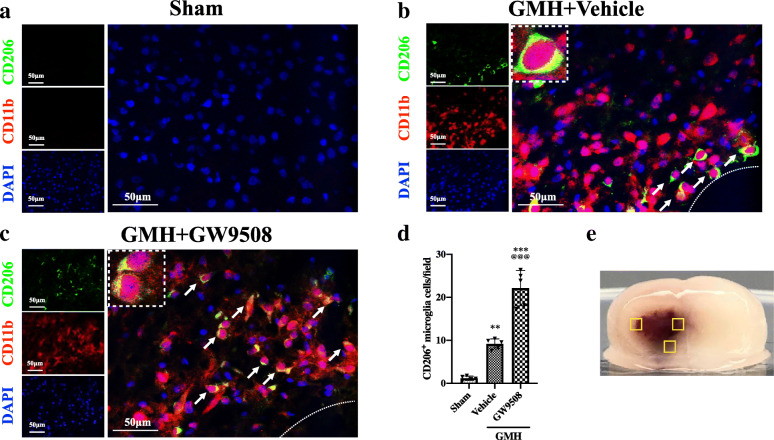
GW9508 upregulated the number of M2 microglia surround hematoma at 24 h after GMH. CD206 and CD11b were used to detect the number of M2 microglia in sham (**a**), GMH (**b**) and GMH + GW9508 groups (**c**). **d** The number of CD206-positive microglia per field. The error bars represent the mean ± SD. n = 5 per group, three regions surrounding the hematoma (**e**) were analyzed in each rat. **p < 0.01 and ***p < 0.001 vs. sham, ^@@@^*p* < 0.001 vs GMH + vehicle. Note: peri-hematoma region is below the white broken line

Double immunofluorescence was used to detect M1 and M2 microglia populations. M1 microglia were quantified by the number of CD16- and CD11b-positive cells in the following three groups: sham, GMH + vehicle, and GMH + GW9508 treatment groups. The result demonstrated that the number of M1 microglia was significantly increased in GMH + vehicle group when compared to the sham group at 24 h after GMH (Fig. 5a, b, and d). However, the number of M1 microglia was decreased in the GMH + GW9508 treatment group when compared to the GMH + vehicle group at 24 h after GMH (Fig. 5c, d). The number of M2 microglia (CD206^+^/CD11b^+^ cells) in the GMH + vehicle group was significantly increased when compared with the sham group at 24 h after GMH (Fig. 6a, b, and d). Meanwhile, the number of M2 microglia significantly increased in the GMH+GW9508 treatment group compared to both the GMH + vehicle and sham group at 24 h after GMH (Fig. 6c, d). The results suggested that GW9508 treatment reduced M1 microglia populations and increased M2 microglia populations at 24 h after GMH.

### GW9508 treatment ameliorated long-term neurobehavioral impairment after GMH

**Fig. 7:**
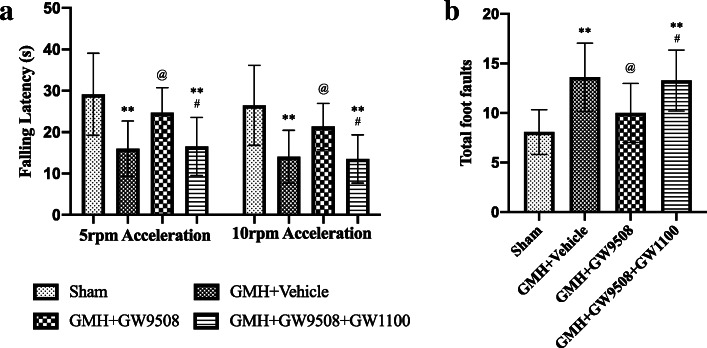
GW9508 treatment improved motor function but GW1100 reversed the protective function at 28 days after GMH. GW9508 improved GMH pups’ motor function but GW1100 reversed the protective function evaluated by rotarod test (**a**) and foot fault (**b**). **p < 0.01 vs Sham, ^@^*p* < 0.05 vs GMH + vehicle, ^#^*p* < 0.05 vs GMH + GW9508, one-way ANOVA, Tukey’s test, n = 15

Rotarod and foot fault tests were used to assesses motor coordination. The GMH + vehicle group demonstrated shorter falling latency in both 5 rpm and 10 rpm accelerating velocity experiments compared to the sham group at 28 days after GMH (Fig. 7a). GW9508 treatment improved the falling latency of GMH animals when compared to the GMH + vehicle group (Fig. 7a). However, the improvements induced by GW9508 treatment were reversed by GW1100 (a selective GPR40 antagonist) (Fig. 7a). The foot fault test demonstrated that rats in the vehicle group had a higher number of total foot faults when compared with the GW9508 treatment group (Fig. 7b). The effects of the treatment were reversed by GW1100 (Fig. 7b).

**Fig. 8:**
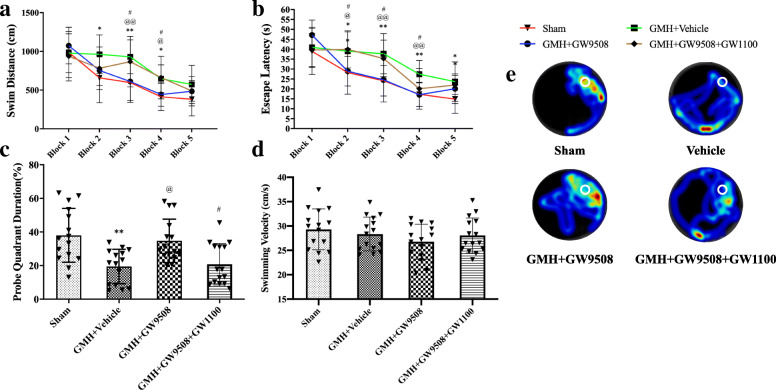
GW9508 treatment improved memory function but GW1100 reverse the protective function at 21–28 days after GMH. GW9508 administration significantly improved memory function as shown by less **a** swimming distance, **b** escape latency, and **c** percentage of time in the probe quadrant in Morris water maze test, but the swim speed (**d**) showed no significant difference between the four groups. However, GW1100 reverse the protective function. **e** Representative thermal imaging of the probe trial. The white circles indicate the positions of the probe platform. *p < 0.05 and **p < 0.01vs Sham, ^@^*p* < 0.05 and ^@@^*p* < 0.01 vs GMH + vehicle, ^#^*p* < 0.05 vs. GMH + GW9508. n = 15

The Morris water maze was used to test spatial and reference memory after GMH. The water maze test demonstrated that vehicle animals had prolonged escape latency and increased swim distance (Fig. 8a, b) and spent significantly less time in the target quadrant in the probe trial (Fig. 8c) when compared with the sham group at 23–27 days after GMH. GMH rats treated with GW9508 had significantly improved spatial memory and learning, which resulted in decreased escape latency, swimming distance, and increase in time spent in the probe quadrant when compared to the GMH+ vehicle group (Fig. 8a, b, c, and e). However, the beneficial impact of GW9508 was reversed by GW1100 (Fig. 8a, b, c, and e). No significant difference was seen in the average swim speed between all three groups (Fig. 8d).

### GW9508 attenuated ventriculomegaly and brain atrophy at day 28 after GMH

**Fig. 9:**
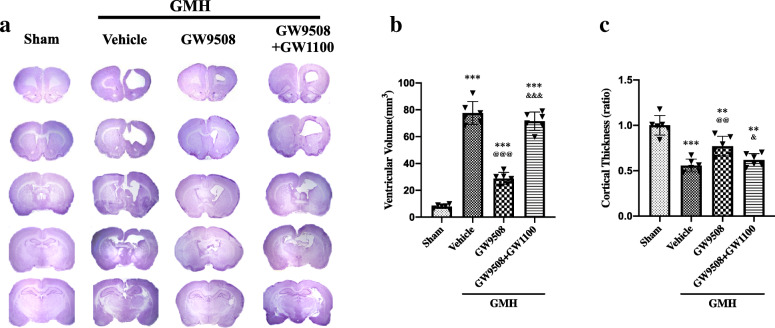
GW9508 attenuated ventriculomegaly and brain atrophy on day 28 after GMH, but reversed by GW1100. GW9508 administration reduced ventricular volume (**a** and **b**) and increased relative cortical thickness (**c**) significantly, but can be reversed by GW1100. ***p < 0.001 vs Sham, ^@@^*p* < 0.01 and ^@@@^*p* < 0.001 vs GMH + vehicle, ^&^*p* < 0.05 and ^&&&^*p* < 0.001vs GMH + GW9508, one-way ANOVA, Tukey’s test, n = 6

Nissl staining was performed to assess ventricular dilation and brain atrophy at 28 days after GMH. Ventricular volume was significantly increased in the vehicle and GW9508 + GW1100 groups, which was significantly reduced in the GW9508 treatment group (Fig. 9a, b). The cortical thickness was significantly decreased in the vehicle and GW9508 + GW1100 group, whereas the GW9508 treatment significantly reduced cortical tissue loss (Fig. 9a, c).

### GPR40 CRISPR, PAK4 CRISPR, and KDM6B CRISPR downregulated the expression of GPR40, PAK4, and KDM6B after GMH

**Fig. 10:**
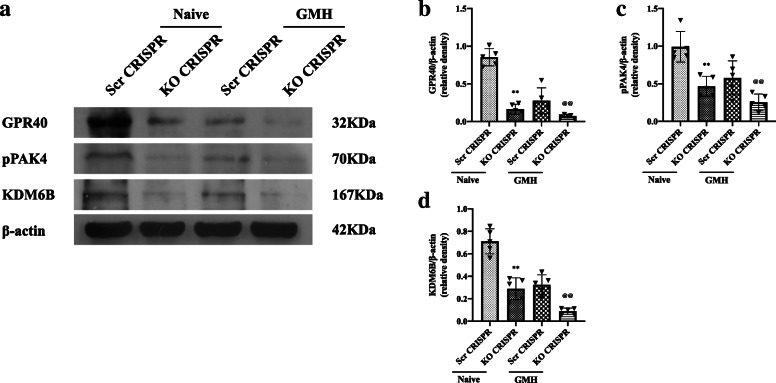
GPR40 CRISPR, PAK4 CRISPR, or KDM6B CRISPR downregulated the expression of GPR40, p-PAK4, or KDM6B after GMH. The representative bands and densitometric quantification of GPR40, p-PAK4, and KDM6B revealed the efficacy of CRISPR knockout in naive and GMH rats, n = 5 per group. Vehicle: 10% dimethyl sulfide. Data was represented as mean ± SD. *p < 0.05 vs. naive group + scr CRISPR group; ^&^*p* < 0.05 vs. GMH + scr CRISPR group; one-way ANOVA, Tukey’s post hoc test; scr CRISPR, scrambled CRISPR; KO CRISPR, knockout CRISPR

Intraventricular injection of GPR40 K.O CRISPR, PAK4 K.O CRISPR, or KDM6B K.O CRISPR was given at 48 h prior GMH. CRISPRs significantly reduced the endogenous expression of GPR40, PAK4, or KDM6B in naive and GMH animals (Fig. 10). These results demonstrated the knockout efficacy of GPR40, PAK4, or KDM6B CRISPRs in the present study.

### Activation of GPR40 via GW9508 attenuated inflammation and mediated microglia polarization via the PAK4/CREB/KDM6B signaling pathway at 24 h after GMH

**Fig. 11:**
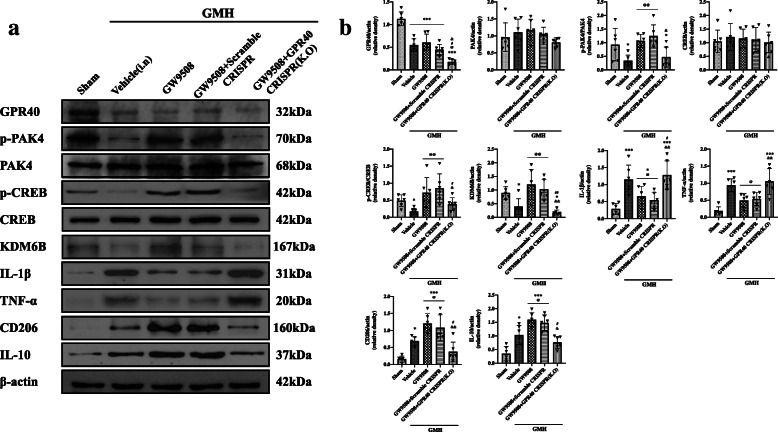
GPR40 K.O CRISPR abolished the beneficial effects of GW9508 at 24 h after GMH. **a**, **b** The representative bands and quantification of GPR40, PAK4, p-PAK4, CREB, p-CREB, KDM6B, IL-1β, TNF-α, CD206, and IL-10. Data was represented as mean ± SD (n = 6 per group). One-way ANOVA was used followed by Tukey’s HSD post hoc test and Holm-Bonferroni correction. *p < 0.05, **p < 0.01, and ***p < 0.001 vs. Sham group; ^@^*p* < 0.05, ^@@^*p* < 0.01 vs. GMH + vehicle group; ^&^*p* < 0.05 and ^&&^*p* < 0.01 vs. GMH + GW9508, ^#^*p* < 0.05 vs. GMH + GW9508 + scramble CRISPR group. Vehicle, 10% dimethyl sulfide

**Fig. 12:**
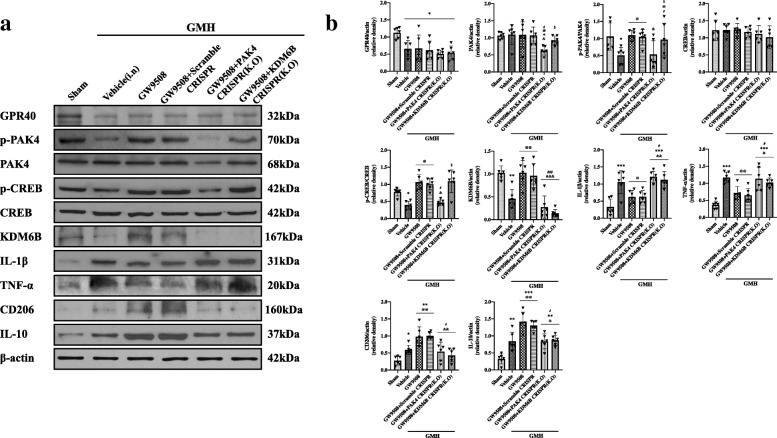
PAK4 and KDM6B K.O CRISPR abolished the beneficial effects of GW9508 at 24 h after GMH. **a**, **b** The representative bands and quantification of GPR40, PAK4, p-PAK4, CREB, p-CREB, KDM6B, IL-1β, TNF-α, CD206, and IL-10. Data was represented as mean ± SD (n = 6 per group). One-way ANOVA was used followed by Tukey’s HSD post hoc test and Holm-Bonferroni correction. *p < 0.05, **p < 0.01, and ***p < 0.001 vs. Sham group; ^@^*p* < 0.05, ^@@^*p* < 0.01 vs. GMH + vehicle group; ^&^*p* < 0.05, ^&&^*p* < 0.01 and ^&&&^*p* < 0.001 vs. GMH + GW9508; ^#^*p* < 0.05 and ^###^*p* < 0.001 vs. GMH + GW9508 + scramble CRISPR group; ^$^*p* < 0.05 vs. GMH + GW9508 + PAK4 K.O CRISPR group. Vehicle, 10% dimethyl sulfide

Western blots demonstrated that GMH significantly decreased the expression of GPR40, p-PAK4, p-CREB, and KDM6B, whereas GMH increased the level of IL-1β, TNF-α, CD206, and IL-10 when compared with the sham group (Figs. 11 and 12). The activation of GPR40 by GW9508 treatment significantly increased the expression of p-PAK4, p-CREB, KDM6B, CD206, and IL-10, which was also met with a decrease in IL-1β and TNF-α expression when compared to the GMH + vehicle group (Figs. 11 and 12). GPR40 K.O CRISPR significantly decreased the expression of GPR40, p-PAK4, p-CREB, KDM6B, CD206, and IL-10 and increased the expression of IL-1β and TNF-α when compared to the GMH + GW9508 group and GMH + GW9508 + scramble CRISPR group (Fig. 11a, b). PAK4 K.O CRISPR significantly decreased the expression of p-PAK4, p-CREB, KDM6B, CD206, and IL-10 and significantly increased the protein level of IL-1β and TNF-α compared to GMH + GW9508 group and GMH + GW9508 + scramble CRISPR group (Fig. 12a, b). Additionally, KDM6B K.O CRISPR significantly downregulated the protein level of KDM6B, CD206, and IL-10 and increased the protein level of IL-1β and TNF-α compared to the GMH + GW9508 group and GMH + GW9508 + scramble CRISPR group (Fig. 12a, b). The results suggest that the PAK4/CREB/KDM6B signaling pathway plays a significant role in the attenuation of neuroinflammation and modifying microglia polarization through the activation of GPR40 after GMH.

## Discussion

In this present study, we investigated the effect of GPR40 agonism via GW9508 on neuroinflammation and its underlying signaling mechanism after GMH. The novel findings of this study are as follows: (1) the expression of GPR40, p-PAK4, and KDM6B decreased at 3 h and reached the lowest level of expression at 24 h after GMH; the expression of M1 marker CD16 was significantly upregulated at 3 h after GMH, while M2 marker CD206 expression had a delayed increase in expression after GMH; (2) GPR40 and KDM6B were found to be expressed in neurons, astrocytes, and microglia at 24 h after GMH; (3) GW9508 at a dose of 2.5 mg/kg improved neurological outcomes at 24 h after GMH; (4) GW9508 attenuated neuroinflammation and decreased M1 and increased M2 microglia populations at 24 h after GMH; (5) GW9508 improved long-term neurobehavior impairments and preserved brain morphology brought on by GMH; (6) and lastly, GPR40 agonism attenuated neuroinflammation and regulated microglia polarization via the PAK4/CREB/KDM6B signaling pathway.

GPR40, also known as free fatty acid receptor 1 (FFAR1), has been shown to be highly expressed in microglia, astrocytes, and neurons in the CNS [17]. Activation of GPR40 has been shown to attenuate the production of critical cytokines and chemokines [46]. The anti-inflammatory effects of GPR40 activation may be attributed to the inhibition of activated microglia (M1) populations through a GPR40-dependent manner. Current literature suggests that GPR40 agonisms play a role in the reduction in glial activation, which reduced subsequent neuronal damage in AD patients [47]. Additionally, intracerebroventricular treatment of GW9508, GPR40 agonist, reduced the expression of inflammatory genes in the hypothalamus and exerted neuroprotective effects after acute cerebral infarction [22, 48]. In the present study, we are the first to demonstrate that GMH decreased the expression of endogenous GPR40 in the neonatal CNS. Furthermore, double immunofluorescence staining showed that GPR40 was co-localized in neurons, astrocytes, and microglia cells in the neonatal brain.

KDM6B (JMJD3) is one of two epigenetic modifiers responsible for the enzymatic removal of the repressive chromatin mark histone H3 lysine 27 trimethylation (H3K27me3). The demethylation of H3K27me3 is catalyzed by KDM6B which acts as a response gene to inflammatory cytokines and regulates macrophage polarization [49]. Suppression of KDM6B in microglia resulted in the decrease of M2 populations and was met with an exponential increase in M1 microglial populations, thereby increasing the inflammatory response which led to an extensive neuronal death [28]. An increase of microglial KDM6B expression was shown to attenuate inflammation and promoted anti-inflammatory effects of M2 after hemoglobin exposure, thereby suppressing inflammation in CNS injury models [27, 50]. In this present study, we are the first to demonstrate that GMH decreased the expression of endogenous KDM6B level in the brain. Meanwhile, double immunofluorescence staining showed that KDM6B was co-localized in neurons, astrocytes, and microglia cells.

Microglia are a unique cell type of immune/myeloid cells in the central nervous system [51]. Studies have shown that microglia are activated within minutes after CNS injury [52]. Current research indicates that microglia in the central nervous system are heterogeneous and can be widely present in polarized phenotypes [53]. The two phenotypes of interest are M1 microglia which mediate the production of inflammatory cytokines and alternatively activated M2 microglia which are characterized as anti-inflammatory modulators [54]. Our previous publications demonstrated that M2 microglia have been attributed to the improvement of both short- and long-term neurobehavioral outcomes after GMH [55]. Flores et al. demonstrated that the upregulation of CD36 in macrophages and microglia increased M2 microglia polarization and was critical for the enhancement of hematoma resolution, which improved overall outcomes [3]. Zhang et al. reported that rh-Chemerin attenuated GMH-induced neuroinflammation through M2 microglia polarization after GMH [30]. Our results demonstrated a significant increase in the number of CD206^+^ cells and decreased CD16^+^ cells in GW9508-treated GMH pups compared to the vehicle group. Our data suggest that the anti-inflammatory effects of GPR40 activation are attributed through microglia polarization.

We further explored the molecular mechanisms underlying the anti-inflammatory effects of GPR40 activation after GMH. The p21-activated kinases (PAKs) are a family of protein serine/threonine kinases [56]. Initial studies on PAK4 have revealed its role in the reorganization of the actin cytoskeleton, neuronal development, and regulation of axonal outgrowth in neural progenitor cells. Previous studies indicated that the activation of the GPR40 enhanced PAK4 S474 phosphorylation [23]. CAMP-response element-binding (CREB) becomes activated by PAK4 phosphorylation, which in turn induces gene expression of KDM6B, which mediates inflammatory gene expression and regulates microglia polarization [24, 25]. In our present study, we demonstrated that the GPR40 activation by GW9508 significantly upregulated the expression of p-PAK4, p-CREB, KDM6B, CD206, and IL-10, but downregulated the protein levels of IL-1β and TNF-α in the neonatal CNS at 24 h post-GMH. The knockdown of GPR40 decreased GPR40 expression and downstream signal molecules p-PAK4, p-CREB, KDM6B, CD206, and IL-10, while the knockdown resulted in the increased expression of IL-1β and TNF-α. Furthermore, the knockdown of PAK4 significantly decreased the expression of p-PAK4, p-CREB, KDM6B, CD206, and IL-10, but increased the protein expression of IL-1β and TNF-α. Lastly, the knockdown of KDM6B significantly reduced the protein level of KDM6B, CD206, and IL-10 and upregulated the protein level of IL-1β and TNF-α. Our results indicate that GPR40 activation upregulates the KDM6B partly via the PAK4/CREB signaling pathway, demonstrating its role in anti-inflammation and microglia polarization after GMH.

The present study has several limitations. First, previous studies and our results revealed that GPR40 was also found to be localized in astrocytes and neurons. Yet this manuscript only focused on the mechanism of action of GPR40 in microglia cells, and further studies need to be conducted to establish the role of GPR40 on other cell types. Second, we verified a novel mechanism of the PAK4/CREB/KDM6B signaling pathway in the anti-inflammatory effect of GPR40 activation after GMH. However, other signaling pathways involved in the anti-inflammatory process such as AMPK/PLC/IP3 and TLR4/NF-κB as well as ARRB2/NLRP3 need to be elucidated in future studies after GMH [57–59]. Third, although the collagenase injection model is a well-established and a consistent model to mimic the GMH pathophysiology, collagenase may exacerbate the inflammatory process and future studies need to be conducted to determine if collagenase significantly plays a role in the inflammatory process when modeling GMH [60]. Collagenase contribution to increased inflammation after GMH may account for the development of ventricular dilation. Therefore, it may differ from the cerebrovascular mechanism of a spontaneous cerebral hemorrhage in premature human newborns [61].

## Conclusions

In conclusion, this study is the first to demonstrate that GPR40 agonism via GW9508 ameliorated neurobehavioral impairments by attenuating neuroinflammation and regulating microglia polarization after GMH in neonatal rats. The anti-inflammatory and microglia polarization effects of GPR40 agonism were mediated, at least in part, through the PAK4/CREB/KDM6B signaling pathway. Therefore, GPR40 demonstrates promise in being a potential non-invasive target to attenuate neuroinflammation after GMH.

## Data Availability

The datasets used and/or analyzed in the current study are available from the corresponding authors on request.

## Abbreviations

BBB: Blood-brain barrier
BLVRA: Biliverdin reductase A
CNS: Central nervous system
CREB: CAMP-response element-binding
CRISPR: Clustered regularly interspaced short palindromic repeats
EPA: Eicosapentaenoic acid
FFA: Free fatty acid
FIZZ1: Hypoxia-induced mitogenic factor
GMH: Germinal matrix hemorrhage
GPR40: The orphan G-protein-coupled receptor 40
IL-10: Interleukin-10
IL-1β: Interleukin-1β
IL-12: Interleukin-12
iNOS: Inducible nitric oxide synthase
KDM6B: Histone H3 lysine 27 (H3K27) demethylase
PAK4: p21-Activated kinases4
TNF-α: Tumor necrosis factor-α
Ym1: Mouse chitinase 3-like 3
